# Folate Deficiency Decreases Apoptosis of Endometrium Decidual Cells in Pregnant Mice via the Mitochondrial Pathway

**DOI:** 10.3390/nu7031916

**Published:** 2015-03-13

**Authors:** Xing Gui Liao, Yan Li Li, Ru Fei Gao, Yan Qing Geng, Xue Mei Chen, Xue Qing Liu, Yu Bin Ding, Xin Yi Mu, Ying Xiong Wang, Jun Lin He

**Affiliations:** Laboratory of Reproductive Biology, School of Public Health, Chongqing Medical University, Chongqing, 400016, China; E-Mails: xingjin19880304@163.com (X.G.L.); colourful0324@yeah.net (Y.L.L.); gao_ru_fei@163.com (R.F.G.); aqing000@163.com (Y.Q.G.); shirly-cxm@163.com (X.M.C.); a68733172@online.cq.cn (X.Q.L.); dingyb@gmail.com (Y.B.D.); anniemachine@163.com (X.Y.M.); wyx61221@aliyun.com (Y.X.W.)

**Keywords:** folate deficiency, decidual cells, apoptosis

## Abstract

It is well known that maternal folate deficiency results in adverse pregnancy outcomes. In addition to aspects in embryonic development, maternal uterine receptivity and the decidualization of stromal cells is also very important for a successful pregnancy. In this study, we focused on endometrium decidualization and investigated whether apoptosis, which is essential for decidualization, was impaired. Flow cytometry and TUNEL detection revealed that apoptosis of mouse endometrium decidual cells was suppressed in the dietary folate-deficient group on Days 7 and 8 of pregnancy (Day 1 = vaginal plug) when decidua regression is initiated. The endometrium decidual tissue of the folate deficiency group expressed less Bax compared to the normal diet group while they had nearly equal expression of Bcl2 protein. Further examination revealed that the mitochondrial transmembrane potential (ΔΨm) decreased, and the fluorescence of diffuse cytoplasmic cytochrome c protein was detected using laser confocal microscopy in normal decidual cells. However, no corresponding changes were observed in the folate-deficient group. Western blotting analyses confirmed that more cytochrome c was released from mitochondria in normal decidual cells. Taken together, these results demonstrated that folate deficiency could inhibit apoptosis of decidual cells via the mitochondrial apoptosis pathway, thereby restraining decidualization of the endometrium and further impairing pregnancy.

## 1. Introduction

Numerous studies have examined the effect of folate deficiency on birth defects, and folate deficiency has been acknowledged to be a vital risk factor of neural tube defects (NTD) [1]. Several studies on the effect of folate deficiency on reproduction have mainly focused on fetal development, and the adverse effects of folate deficiency on embryonic development have been well confirmed [2,3]. Successful gestation requires not only normal development of the embryo itself but also a suitable maternal endometrium, such as the establishment of uterine receptivity and decidualization. Our previous study revealed that there was no effect of folate deficiency on embryo implantation, and both the expression of uterine receptivity marker genes and the number of implantation sites showed no significant difference between the folate deficiency group and control group [4]. However, the outcomes of the folate-deficient pregnant mice were not favorable, and we found a lower birth rate and more embryo loss in folate-deficient pregnant mice (unpublished data). Thus, it remains an outstanding question of whether folate deficiency plays a role in the process of decidualization after embryo implantation. In this study, we investigated maternal uterine endometrium decidualization under folate-deficient conditions.

During early pregnancy in mice, the onset of embryo implantation occurs in the receptive uterus, followed by a transformation of stromal cells to decidual cells in the morning on day 5 of pregnancy (Day 1 = vaginal plug) [5]; this process is termed decidualization. Apoptosis occurs following proliferation and differentiation of stromal cells in the decidual zone after implantation [6]. Numerous studies have demonstrated that involution of the pregnant uterus begins with luminal epithelial cells and subsequently spreads throughout the anti-mesometrial zone. Finally, the mesometrial decidual cells undergo degeneration [7]. On the basis that cell elimination in the luminal epithelium and mature decidua also occurs in artificially induced decidualization cells, the apoptosis of uterine cells in pregnant mice is thought to be due to an intrinsic cellular pathway [8]. The apoptosis of endometrium decidual cells has attracted some research attention. Abrahamsohn first investigated the morphological aspects of apoptosis by analyzing the ultrastructure of mouse decidual cells and described the initial period of involution on Day 7 (D7) and Day 8 (D8) of pregnancy [9]. Sima Katz *et al.* [7] subsequently observed the accumulation of clumps of chromatin, dilation of the cisterna and endoplasmic reticulum, changes in the morphology to a sphere and loss of plasma membrane and suggested cell death of the decidua as a type of programmed cell death (apoptosis). Proteins involved in the apoptosis of decidual cells may be multitudinous and the mechanism undetermined, while a shift in Bcl2 family protein expression plays a vital role in initiating apoptosis of the decidualized mesometrium [10]. Kamil C. *et al.* [11,12] showed that Bcl2 family members decide the fate of decidual cells *in vivo* and *in vitro*. Thus, the involution of uterine cells is deemed to be the terminal step of the cell differentiation process associated with decidualization due to the same spatial sequence of stromal transformation and apoptosis of decidual cells [11].

The effect of folate deficiency on apoptosis varies in different tissues or cells and involves a variety of molecular mechanisms [13,14,15,16,17]. Due to the key role of apoptosis and decidualization in the development and remodeling of the uterine endometrium after embryo implantation and the fact that the effect of folate deficiency on apoptosis of decidual cells remains unknown, the purpose of this study is to elucidate the effect of folate deficiency on apoptosis of the uterine endometrium decidual cells and the related potential mechanism. These results would provide critical clues to explain the adverse pregnancy outcomes resulting from folate deficiency.

## 2. Experimental Section

### 2.1. Ethical Approval

All animal procedures were approved by the Ethics Committee of Chongqing Medical University (20110016) on 21 October 2011.

### 2.2. Animals and Tissue Collection

The folate-deficient pregnant mouse model was established according to the method of a previous report [4]. Briefly, six- to eight-week-old NIH mice approved for experimental use by the Laboratory Animal Center of Chongqing Medical University (NO. 20110016) were housed in a specific pathogen-free animal room under a controlled photoperiod (12 h light/12 h darkness). We randomly divided the female mice into two groups with 80 mice in each group. The folate-deficient group was fed a diet containing no folate, and the control group was fed a normal diet. After five weeks, estrus mice were selected to mate with mature healthy males of the same strain. The day in which a vaginal plug was found after mating was considered to be the first day of pregnancy (D1). Uterine endometrial tissue on D7 and D8 was collected on ice and quickly stored at −80 °C for further analyses.

### 2.3. Detection of Serum Folate Levels

Serum folate levels of pregnant mice were detected using an electro-chemiluminescence immunoassay as previously described [18]. Briefly, the serum of both control group and folate-deficient group pregnant mice were collected. The new capillary was preconditioned by flushing with 1M NaOH for 30 min before the first use. Samples were then injected into the capillary by hydrodynamic flow at a height differential of 20 cm for 10 s. Running voltages was 16 Kv. Electrophoresis electrolyte was 0.8 mM luminol in 35 mM borate buffer (pH 9.4). The chemiluminescence emission was collected by a photo multiplier tube (PMT, R374 equipped with a C1556-50 DA-type socket assembly, Hamamatsu, Shizuoka, Japan), and recorded and processed with an IBM compatible computer using in-house written software.

### 2.4. Transmission Electron Microscopy

The antimesometrial region was chosen as the subject of study. Briefly, the antimesometrial decidua and myometrium of pregnant mice on D7 and D8 were dissected under a stereomicroscope, immediately post-fixed in osmium tetroxide and embedded in Araldite. Ultrathin sections were cut transversely along the long axis of the uterus, stained with 2% aqueous uranyl acetate and 0.5% lead citrate. Examinations of the sections were performed by a professional operator using a TEM Hitachi-7500.

### 2.5. Isolation and Culture of Primary Decidua Cells

Twelve pregnant mice on D7 and D8 from the control diet and the folate-deficient diet group, respectively, were sacrificed, and the endometrial tissues were immediately removed and placed in PBS under aseptic conditions. After three washes with PBS to remove excess blood, the embryos were dissected under a stereomicroscope. The decidual tissue was finely minced and digested with enzyme Ι (containing 0.6% dispase and 2.5% trypsin) at 4 °C for 1 h, followed by room temperature for 1 h, and an additional 10 min at 37 °C for preliminary digestion. After further digestion with 0.05% collagenase, a 70-m cell strainer was used to sieve the supernatant, and the decidual cells were collected by centrifugation. The cell precipitation was resuspended with a complete medium and cultured in a flask in a 5% CO2 incubator at 37 °C. After 1 h of incubation, the culture medium was removed to eliminate the non-adherent cells and a new medium was added into the culture flask, which would ensure the purity of the primary decidual cells. Decidual cells from different groups were cultured in corresponding complete medium (Roswell Park Memorial Institute 1640, Sigma, St. Louis, MO, USA), both containing 10% fetal bovine serum (Sigma, St. Louis, MO, USA) and supplemented with 100 mg/mL streptomycin and 100 U/mL penicillin). The decidual cells showed a fusiform or round morphology with one or more nuclei and were identified by detecting the expression of bone morphogenetic protein 2 (BMP2) using immunofluorescence.

### 2.6. Real-Time PCR

Total RNA was extracted from mouse decidual tissue of normal diet-fed and folate-deficient diet-fed pregnant mice (D7 and D8) with TRIzol reagent (TaKaRa, Dalian, China) and reverse-transcribed into cDNA using the PrimeScriptTM RT Reagent Kit (TaKaRa, Dalian, China) according to the manufacturer’s instructions. The primers used in this study are shown in Supplementary material 1 (see Supplementary Table S1). β-Actin was used as an internal control for standardization. Real-time PCR was performed using the SYBR Premix Ex TaqTM Kit (TaKaRa, Dalian, China) on a BIO-RAD iQ5 Multicolor Real-Time PCR Detection System. Experiments were performed in triplicate. Data obtained from real-time PCR were analyzed using the 2-^ΔΔCt^ method, and statistical analysis was performed using Prism Graphpad 5.0.

### 2.7. TUNEL Assay

Terminal deoxynucleotidyl transferase-mediated dUTP nick end labeling (TUNEL) assays were performed using a TUNEL kit (Roche, Mannheim, Germany) according to the manufacturer’s instructions. The uterine tissue sections were pretreated with 20 μg/mL Proteinase K for 15 min at 37 °C, washed in PBS, and then incubated with TUNEL reaction mixture (label solution and enzyme solution) for 1 h at 37 °C. After rinsing the sections three times in PBS for 5 min, the sections were observed under a confocal fluorescence-microscope system. Green fluorescence indicates positive cells.

### 2.8. Western Blotting Analysis

A tissue protein extraction kit (Beyotime, Shanghai, China) was used for protein preparation. Total and cytosolic proteins were extracted from the tissue samples of folate deficiency and control group pregnant mice (*n =* 6 each) and then boiled in 5× SDS sample loading buffer for 10 min. Equal amounts of total protein (50 g) were separated using 10% sodium dodecyl sulfate-polyacrylamide gel electrophoresis (SDS-PAGE) and transferred onto nitrocellulose membranes (Bio-Rad Laboratories). Membranes were blocked in 5% milk for 1 h at room temperature followed by the appropriate primary antibodies diluted in blocking buffer (4 °C, overnight) as previously described. After several washes, the membranes were incubated with specific secondary antibodies corresponding to the source of primary antibodies for 1 h at room temperature. After 4 washes with PBST (5 min each), the immunoreactive bands were visualized using ChemiDocTM XRS+(Bio-Rad) and chemiluminescence reagents (Millipore, WBKLS0500, Billerica, MA, USA). Densitometry measurements were analyzed using Quantity One v4.4.0 (Bio-Rad Laboratories). Protein expression levels were normalized against β-actin. The following primary antibodies were used: Rabbit polyclonal anti-cytochrome c (Sangon Biotech, AB20521a, Shanghai, China, 1:500 dilution), rabbit monoclonal anti-Bax (Millipore, 04-434, USA, 1:1000 dilution), rabbit polyclonal anti-Bcl2 (Sangon Biotech, AB20521a, Shanghai, China, 1:600 dilution), rabbit polyclonal anti-pro-Caspase3 (Sangon Biotech, AB20074a, Shanghai, China, 1:600 dilution), rabbit polyclonal anti-cleaved-caspase3 (Millipore, AB3623, Billerica, MA, USA, 1:1000 dilution), rabbit polyclonal anti-RELA (phospho-ser276) (Sangon Biotech, AB55005, Shanghai, 1:700 dilution), rabbit polyclonal anti-RELA (Sangon Biotech, AB21030b, Shanghai, China, 1:650 dilution), rabbit polyclonal anti-MAPK1 (Sangon Biotech, AB60317a, Shanghai, China, 1:700 dilution), goat monoclonal anti-HOXA10 (Santa Cruz, Dallas, TX, USA, 1:1000 dilution) rabbit polyclonal anti-BMP2 (Millipore, ABN303, Billerica, MA, USA, 1:1000 dilution), mouse monoclonal anti-MMP2 (Millipore, MAB3308, Billerica, MA, USA, 1:1000 dilution), and rabbit monoclonal anti-MMP9 (Abcam, ab137867, Cambridge, MA, USA, 1:1200 dilution).

### 2.9. Immunohistochemistry

The tissues were fixed in 4% paraformaldehyde, dehydrated by increasing concentrations of alcohol, and subsequently embedded in paraffin. Sections (5 μm) were prepared for further study. For immunohistochemistry, antigen retrieval was performed in sodium citrate buffer for 10 min at room temperature, followed by 15 min at 100 °C in a microwave oven. Endogenous peroxidase was inhibited by incubation with 3% hydrogen peroxide for 10 min at room temperature. The sections were blocked in 10% normal goat serum for 30 min at 37 °C, incubated with primary antibody at 4 °C overnight, and then incubated with secondary antibody for 30 min at 37 °C followed by streptavidin-conjugated horseradish peroxidase for 30 min at 37 °C. Antibody staining was developed using a diaminobenzidine substrate. The sections were subsequently stained with hematoxylin.

### 2.10. Immunofluorescence and Confocal Fluorescence Microscopy

The release of cytochrome c from mitochondria into the cytoplasm was observed using a confocal fluorescence microscope equipped with an argon-ion laser and He-Cd laser. Decidua cells isolated from the uterine endometrium tissue of pregnant mice (D7 and D8) were cultured on slides (10 mm × 10 mm) for two days with medium changed everyday. After three washes with PBS, the sections were fixed in cold methanol for 15 min at room temperature and blocked with 2% BSA for 1 h at 37 °C, incubated overnight with primary antibodies at 4 °C, and then incubated with fluorescein isothiocyanate (FITC)-labeled rabbit IgG for 1 h at 37 °C in the dark. After incubation with PI (Solarbio, P4170, Beijing, China) and sealing by glycerine (50%), the sections were observed using confocal fluorescence microscopy (MRC-600, Bio-Rad) and inverted epifluorescence microscopy (Nikon TMD-EFQ) at a wavelength of 488 nm. The primary antibodies used in this study were cytochrome c, BAX, and BMP2.

### 2.11. Mitochondria Extraction and Detection of Mitochondrial Transmembrane Potential (ΔΨm)

Isolation of mitochondria from endometrium decidual tissues was performed using the Tissue Mitochondria Isolation Kit (Beyotime, Beijing, China, C3006). The mitochondrial transmembrane potential (ΔΨm) of decidua primary cells isolated from each group was detected using tetrachloro-tetraethylbenzimidazol carbocyanine iodide (JC-1) staining (20 μg/mL) for 35 min at 37 °C. JC-1 fluorescence was observed using confocal fluorescence microscopy as previously described with an excitation wavelength of 490 nm and 510 nm for green fluorescence and red fluorescence, respectively.

### 2.12. Flow Cytometric Evaluation of Apoptosis

Decidua primary cells were harvested, washed and resuspended with culture medium. Cells that have lost membrane integrity will demonstrate red staining (propidium iodide, PI) throughout the nucleus and will thus be easily distinguished as early apoptotic, late apoptotic, or necrotic cells. Samples were incubated at room temperature for 15 min in the dark with Annexin V and PI and then quantitatively analyzed using a FACS Vantage SE flow cytometer.

### 2.13. Statistical Analyses

All experiments were replicated at least three times. The data were analyzed using the Statistical Package for the Social Sciences (SPSS) statistical software (Version 16.0; SPSS Inc., Chicago, IL, USA). Values are expressed as the mean ± SD. Student’s *t*-test was used to analyze differences between groups. Differences were considered significant if *p* < 0.05.

## 3. Results

### 3.1. Folate-Deficient Mice Have a Lower Level of Serum Folate

**Figure 1:**
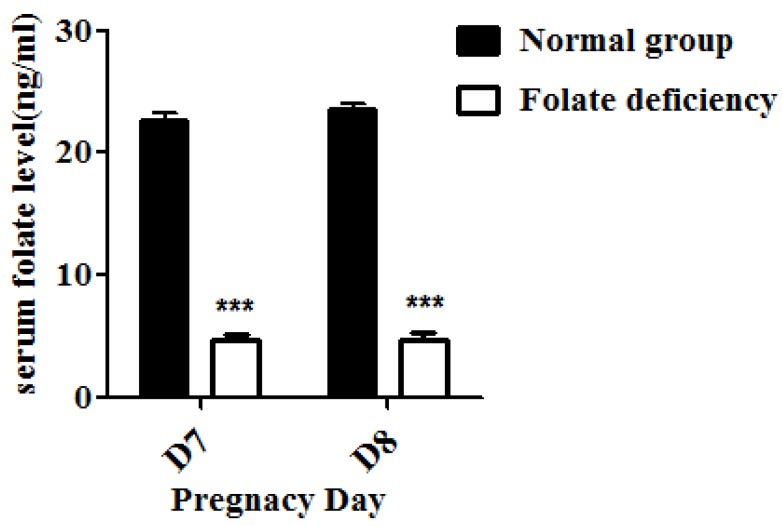
Serum folate concentrations detected using an electro-chemiluminescence immunoassay; lower serum folate concentrations were observed following folate deficiency treatment, *** *p* < 0.001.

The serum folate concentration was detected to validate the folate-deficient mouse model. Serum folate concentrations were obtained from mice in the folate-deficient group and were significantly lower compared to the control group (4.83 ± 2.046 *vs.* 22.75 ± 1.315 ng/mL, *p* < 0.001, *n =* 10, mean ± SEM) (Figure 1), which indicated the successful establishment of the animal model.

### 3.2. Folate Deficiency Reduced Apoptosis of Endometrium Decidual Cells in Pregnant Mice

**Figure 2:**
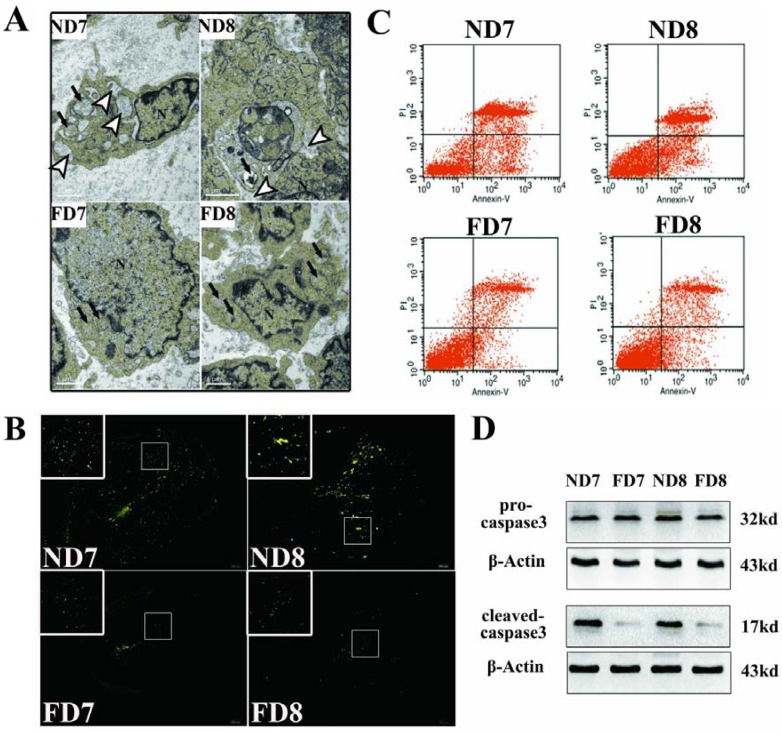
Apoptosis of decidual cells was suppressed in response to folate deficiency treatment. ND7, pregnant mice on the 7th day of pregnancy (d.o.p.) of normal diet group. ND8, pregnant mice on 8th d.o.p. of normal diet group. FD7, pregnant mice on 7th d.o.p. of folate-deficient diet group. FD8, pregnant mice on 8th d.o.p. of folate-deficient diet group. (**A**) Representative images of decidual cells of the two groups as detected using TEM. Arrowhead indicates the dilated endoplasmic reticulum cisternae. Arrows indicate swollen mitochondria (EM. × 15000); (**B**) Representative fluorescence images of TUNEL staining in uterine sections. Green fluorescence indicates positive cells; (**C**) Flow cytometry analysis of decidual cells; (**D**) Western blotting analyses of pro-caspase-3 and cleaved-caspase-3 proteins.

For a morphological comparison of the endometrium decidual cells between folate-deficient mice and control mice, transmission electron microscopy (TEM) was employed to observe differences in the organelles related to programmed cell death. As shown in Figure 2A, decidual cells of mice in the control group exhibited dilation of the perinuclear endoplasmic reticulum cisternae and distended mitochondria, while cells in the folate-deficient mice did not show corresponding changes or showed slight dilation in some cases. To further confirm the effect of folate deficiency on apoptosis in decidual cells, TUNEL and flow cytometry were performed (Figure 2B,C). Compared with the control mice, the number of TUNEL-positive cells was much less in the folate-deficient diet mice, and the results of the flow cytometric analysis showed that in normal D7 and D8 pregnant mice decidual cells, the percentage of early apoptotic cells were 12.80% and 12.79% and the percentages of late apoptotic cells were 60.98% and 48.97%, respectively. While in folate-deficient D7 and D8 pregnant mice decidual cells, early apoptotic cells account for 7.99% and 8.33% and late apoptotic cells account for 22.87% and 17.19%, respectively. This data was consistent with the TUNEL assay. Furthermore, the protein expression of caspase-3, a downstream effector during apoptosis, was significantly downregulated in response to folate deficiency treatment (Figure 2D).

### 3.3. Folate Deficiency Alters the Expression of Bcl2 Family Proteins

**Figure 3:**
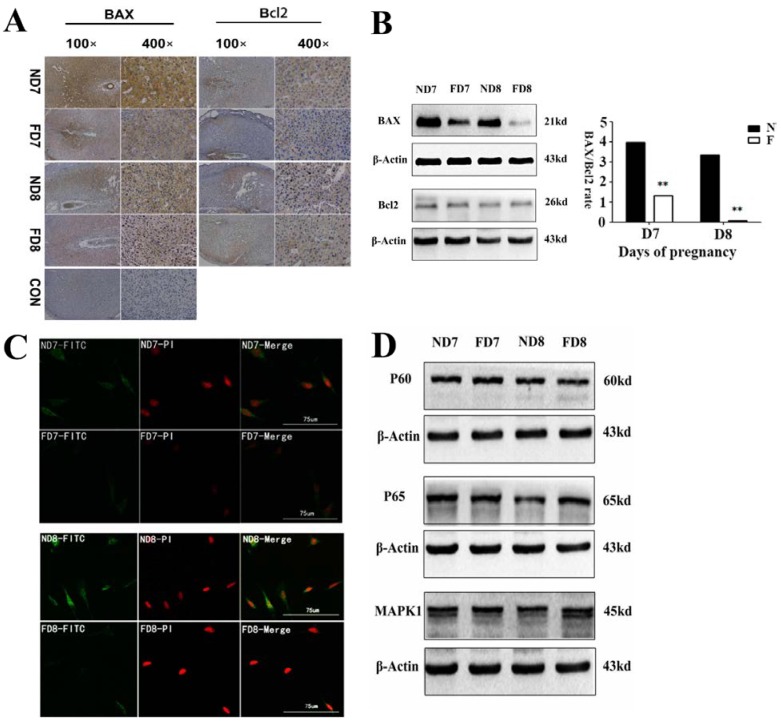
Expression of Bcl2 family proteins in decidual tissues between the folate-deficient group and control group. (**A**) Western blotting analyses of Bax protein and Bcl2 protein. The bar graph on the right shows the Bax/Bcl2 protein ratio analyzed using Quantity One. The value is presented as the mean ± SD from three independent experiments. ** *p* < 0.01; (**B**) Immunohistochemical analysis of Bax and Bcl2 expression in the implantation site decidua on Days 7 and 8 of pregnancy. Brown indicates positive cytoplasmic staining for the localization of immunoreactive Bax and Bcl2 proteins on decidual cells. No immunostaining (NTC) was observed when similar sections were incubated with pre-immune serum. em, embryo; dc, decidual cells; le, luminal epithelial cells; (**C**) Immunofluorescence of Bax protein with decidual cells isolated from decidual tissue on Days 7 and 8 of pregnancy. The folate-deficient group showed a weaker intensity of fluorescence on both days, indicating a lower expression of Bax protein in this group; (**D**) Western blotting analyses of upstream proteins of Bcl2 (P60, P65, MAPK1). All three proteins showed no differences between the folate-deficient and control groups.

Previous studies have confirmed the regulation of Bcl2 family proteins in controlling the involution of endometrium decidual cells. Thus, we proposed that the expression of Bcl2 family proteins may be altered under folate-deficient conditions. Immunohistochemistry studies showed that Bax and Bcl2 proteins are widely distributed in the cytoplasm of decidual cells, and the number of Bax-positive cells was significantly less in folate-deficient mice, while there was no difference in the number of Bcl2-positive cells (Figure 3A). Similarly, western blotting analyses revealed that there were no differences in Bcl2 expression between the folate-deficient and control groups, while the expression of Bax was significantly downregulated after folate deficiency treatment. Thus, the ratio of Bax/Bcl2 was significantly lower in folate-deficient mice (Figure 3B). In addition, the expression of Bax in primary cells was confirmed using immunofluorescence. The fluorescence intensity of Bax protein was much weaker in folate-deficient primary decidual cells (Figure 3C). The identification of decidual cells was shown in Supplementary material 2 (see Supplementary Figure S1). Furthermore, the expression of two upstream proteins (NFκB and MAPK1) of Bcl2 was detected using western blotting analyses. These results showed no obvious differences in the expression of NFκB and MAPK1 between the folate-deficient group and control group (Figure 3D). These data confirmed that folate deficiency suppressed apoptosis of decidual cells by changing the expression of Bcl family proteins.

### 3.4. Folate Deficiency Inhibited Apoptosis via the Mitochondrial Pathway

**Figure 4:**
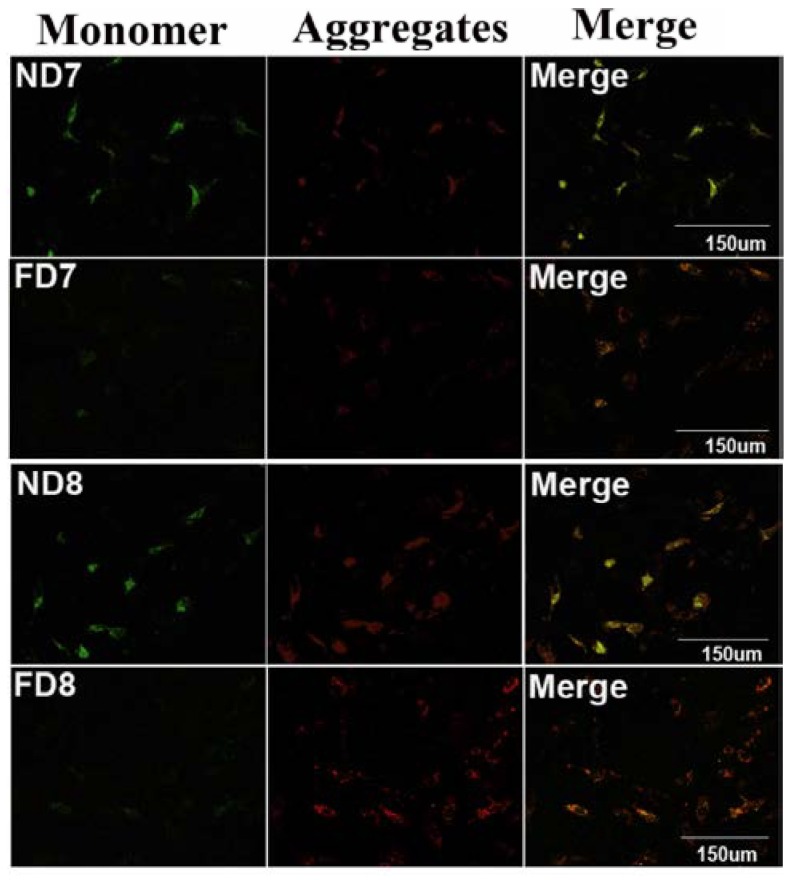
Mitochondrial transmembrane potential (ΔΨm) of mouse endometrium decidual cells did not decrease following folate deficiency treatment (×200). Green fluorescence represents monomeric JC-1 in the cytoplasm (left panel), while red fluorescence represents JC-1 aggregates accumulating in the mitochondria due to a high mitochondrial membrane potential (middle panel). ND7, pregnant mice on 7th d.o.p. of normal group. ND8, pregnant mice on 8th d.o.p. of normal group. FD7, pregnant mice on 7th d.o.p. of folate-deficient group. FD8, pregnant mice on 8th d.o.p. of folate-deficient group.

**Figure 5:**
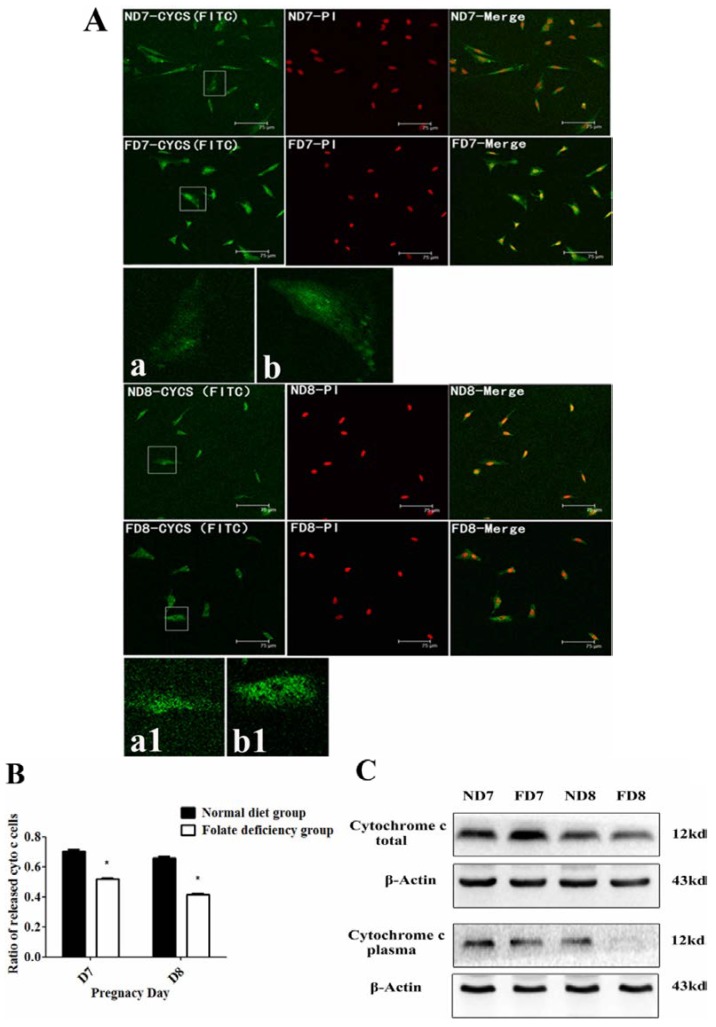
Folate deficiency reduced the cytochrome c release during apoptosis of decidual cells. ND7, pregnant mice on 7th d.o.p. of normal group. ND8, pregnant mice on 8th d.o.p. of normal group. FD7, pregnant mice on 7th d.o.p. of folate-deficient group. FD8, pregnant mice on 8th d.o.p. of folate-deficient group. (**A**) Release of cytochrome c detected using immunofluorescence (×200). Green fluorescence represents cytochrome c. (a/a_1_) and (b/b_1_) represent typical images of cells with different release profiles of cytochrome c in the control group or folate-deficienct group, respectively (×200); (**B**) Percentage of cells with released cytochrome c in total observed cells is presented as the mean ± SD from five different fields. * *p* < 0.05. C, Western blotting analysis of cytochrome c.

The mitochondrial ΔΨm was assessed using a JC-1 fluorescence probe, which revealed that apoptotic primary decidual cells in the control group had decreased mitochondrial membrane potential, while primary decidual cells isolated from mice in the folate-deficient group did not exhibit similar changes (Figure 4). A decreased mitochondrial membrane potential would initiate a downstream “ripple effect,” such as the release of cytochrome c from the intermembrane space of mitochondria. We further compared the release of cytochrome c from mitochondria in primary cells isolated from mice in the two groups using immunofluorescence and confocal fluorescence microscopy. As shown in Figure 5B, the control group showed a higher rate of cells with diffuse cytochrome c distribution, indicating the release of cytochrome c from mitochondria into the cytosol after depolarization. Conversely, decidual cells from the folate-deficient group showed punctate fluorescence, which indicates concentrated cytochrome c in the mitochondria. Western blotting analyses were performed to further confirm the profile of cytochrome c release (Figure 5C). These results demonstrated that the expression of total cytochrome c was similar in both groups, but the tissue in the folate-deficient group contained less cytochrome c in the cytoplasm with the mitochondria excluded. Taken together, these results demonstrated that folate-deficient treatment inhibited the decrease in the mitochondrial membrane potential and the following release of cytochrome c in endometrium decidual cells, thereby suppressing the intrinsic apoptotic pathway.

### 3.5. Folate Deficiency Impairs Decidualization in Mice

**Figure 6:**
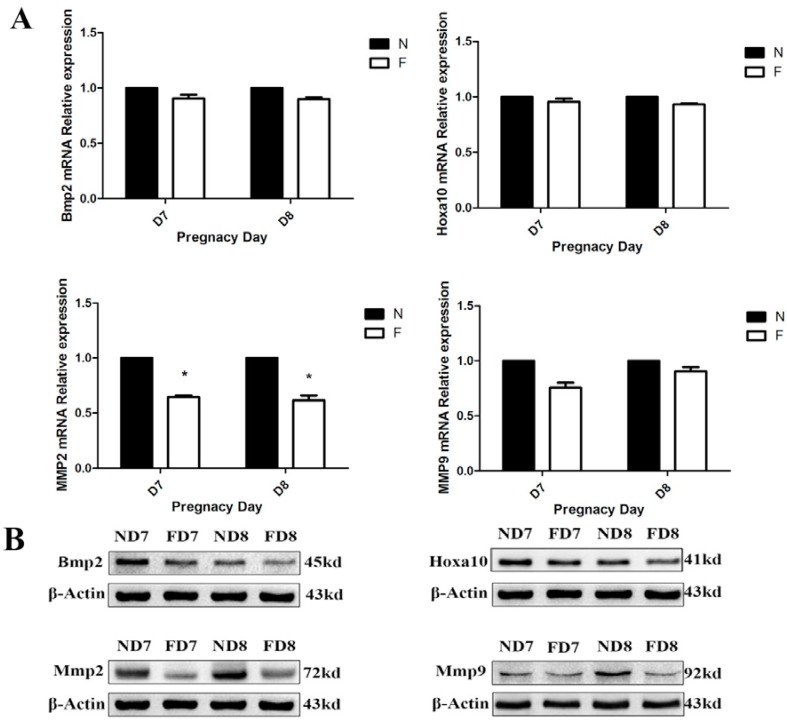

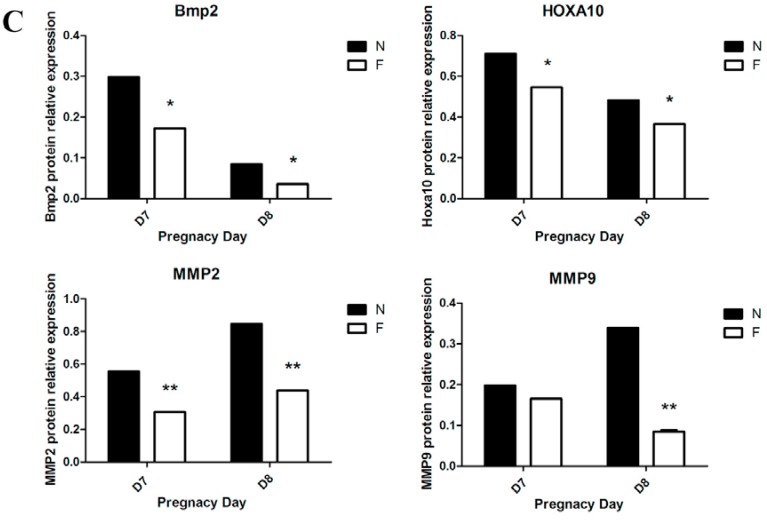
Expression of decidualization marker genes (Bmp2, Hoxa10, MMP2 and MMP9). ND7, pregnant mice on 7th d.o.p. of normal group. ND8, pregnant mice on 8th d.o.p. of normal group. FD7, pregnant mice on 7th d.o.p. of folate-deficient group. FD8, pregnant mice on 8th d.o.p. of folate-deficient group. (**A**) Expression of selected mRNAs detected using Real-time PCR. * *p* < 0.05; (**B**) Western blotting analysis of marker gene proteins; (**C**) Statistical analysis of protein expression from three independent experiments. * *p <* 0.05, ** *p <* 0.01.

As previously described, folate deficiency suppresses the natural apoptosis of decidual cells, which suggests that folate deficiency may have an effect on decidualization. Thus, we analyzed the marker gene expression of endometrium decidualization in mice, including bone morphogenetic protein 2 (BMP2), homeobox A10 (Hoxa10), matrix metalloproteinase 2 (MMP2) and matrix metalloproteinase 9 (MMP9) [19,20]. Real-time PCR results showed that only MMP2 mRNA was significantly decreased following folate deficiency treatment (Figure 6A). Moreover, the expression of BMP2, Hoxa10 and MMP2 proteins was markedly reduced in folate-deficient mice, as revealed using western blotting analyses. The differential expression of MMP9 protein occurred on D8 (Figure 6B,C). Taken together, these data suggested that folate deficiency impaired decidualization in mouse endometrial stromal cells.

## 4. Discussion

It is well known that folate deficiency or depletion induces an unfavorable pregnancy outcome, such as neural tube defects, intrauterine growth retardation, preterm birth [3] and hydrocephalus [21]. Previous studies have mainly focused on the fetus itself in the maternal folate-deficient condition and found multiple congenital abnormalities. As the first molecular event after embryo implantation, decidualization of the endometrium has attracted our attention. It has been confirmed that folate deficiency affects the apoptosis of various cells, and apoptosis of decidual cells is a vital important course during endometrium decidualization both in the mouse and in human. Furthermore, this process is accompanied by proliferation and differentiation of the endometrium stromal cells in mice as well as in humans, ensuring the well-remodeled uterine to receive the gradually-growing embryo [7,12]. Thus, we explored the effect of folate deficiency on the apoptosis of decidual cells, which is a natural process of decidualization.

Transmission electron microscopy is one of the most persuasive methods used to observe cell apoptosis, and thus, it was employed to identify the ultrastructural differences in decidual cells between the control group and folate deficiency group [22]. According to the description provided by Katz *et al.* [7], apoptotic decidual cells showed dilation of the mitochondria and endoplasmic reticulum during involution and then appeared as clumps of chromatin, autophagosomes and heterophagosomes accumulating in the cytoplasm. Consistent with these observations, we found swollen mitochondria and dilated endoplasmic reticulum in decidual cells of normal diet mice, while decidual cells of mice treated with folate deficiency did not show these corresponding characteristics and their organelles appeared nearly non-apoptotic. No evidence of accumulated clumps of chromatin was found in both groups, which may be due to the thin tissue sections (70 nm). We were only able to observe a limited plane of decidual cells, which showed early apoptotic characteristics because apoptosis is a rapid process [23]. In addition, results obtained from the TUNEL assay, flow cytometric analysis and expression of caspase-3 confirmed that folate deficiency reduced apoptosis in decidual cells. In a further study, we found that the expression of Bcl2 family proteins, which regulate apoptosis in decidual cells, changed following folate deficiency treatment. Bcl2 family proteins are critical regulators of programmed cell death via their ability to permeabilize the mitochondrial outer membrane [24,25,26]. Thus, we hypothesized that the mitochondrial pathway might play a role in inhibiting apoptosis due to folate deficiency. The mitochondrial membrane potential was assessed using a fluorescent JC-1 probe with a laser scanning confocal microscope. As a cationic dye, JC-1 converts from a red to a green color if the mitochondrial membrane potential decreases [27]. In polarized (normal) mitochondria, which do not undergo cell apoptosis, JC-1 accumulates and aggregates with a red emission, and when the mitochondria depolarize (loss of mitochondrial membrane potential), JC-1 remains in the cytosol in its monomeric form and fluoresces green [28]. Following depolarization of the mitochondrial outer membrane, the release of cytochrome c is considered to be particularly important in the activation of downstream caspase signaling cascades [23]. Thus, the mitochondrial membrane potential (Δψm) and state of cytochrome c release could be detected. These data confirmed that apoptosis in decidual cells was inhibited by folate deficiency treatment via the mitochondrial pathway.

The effect of folate deficiency on apoptosis varies in different studies. Folate deficiency triggers an oxidative-nitrosative stress-mediated apoptosis in RINm5F Pancreatic Islet β cells [14], while it induces cell apoptosis via a cell cycle arrest mechanism in mouse embryonic stem cells [16]. The NF-κB pathway and Bcl2-related mechanism are also involved in two independent studies [13,29]. However, David Garcia Crespo *et al.* [15] found that folate deficiency decreased apoptosis in normal mouse intestines. This result was not unexpected because previous studies have shown that the effect of folate deficiency is highly cell-specific in gene expression [30] and that the effect may vary between different mouse strains [31]. Thus, we first focused on the effect of folate deficiency on apoptosis of endometrium decidual cells and demonstrated that the natural apoptotic process of decidual cells was suppressed under maternal folate-deficient conditions. Furthermore, decidualization of endometrial stromal cells was impaired in pregnant mice, which was consistent with previous findings indicating that apoptosis is an important component of the decidualization process [32]. However, we proposed that impaired decidualization of the endometrium by folate deficiency may contribute to fetal abnormalities, as previous studies have shown that BMP2, a well-known marker of decidualization, plays an important role in cephalic neural tube closure [33,34]. Thus, further studies are needed to confirm this hypothesis.

This study enriched our awareness that folate deficiency disrupts the proliferation-apoptosis balance and that the mitochondrial apoptosis pathway of endometrial decidual cells was inhibited in folate-deficient pregnant mice. Furthermore, decidualization was impaired. Although future investigations are needed to clarify the molecular mechanisms underlying the effects of folate deficiency on the expression of genes related to the mitochondrial apoptosis pathway and the effect of the folate metabolic pathway on apoptosis of decidual cells, we must first understand the harmful effects of folate deficiency that occur at the time point after embryo implantation. This time point is earlier than the acknowledged period when the neural tube develops, and disrupted decidualization of the uterine endometrium by folate deficiency may have a significant effect on undesirable pregnancy outcomes.

## 5. Conclusions

In this study, we proved that folate deficiency decreased apoptosis of the endometrium decidual cells in pregnant mice. And the disturbed proliferation-apoptosis balance could contribute to impaired decidulization process, which is vital important for embryonic development after embryo implantation. In addition, we speculated that folate deficiency may have an important effect on mitochondrial apoptosis pathway in decidual cells.

## Supplementary Files

Supplementary File 1
