# Supplementary material for: Folate Deficiency Decreases Apoptosis of Endometrium Decidual Cells in Pregnant Mice via the Mitochondrial Pathway
Source: Nutrients. 2015 Mar 13;7(3):1916–32. doi: 10.3390/nu7031916 (PMC4377890; doi:10.3390/nu7031916)
Supplement: Supplementary File 1 [file nutrients-07-01916-s001.doc]

**Supplementary Information**

**Table S1.** Primer sequences of four decidulization-related genes and β-actin.

| **Target Genes** | **Primer Sequence (5’→3’)** | **Product Size** |
| --- | --- | --- |
| Hoxa10 sense | CCAGCCCCTTCAGAAAACAG | 114bp |
| Hoxa10 anti-sense | GCAGCGTTTCTTCCGGC |  |
| MMP2 sense | CTGTCCGCCAAATAAACC | 152 bp |
| MMP2 anti-sense | CCCCGATGCTGATACTGA |  |
| MMP9 sense | CTGTATGGTCGTGGCTCTAA | 181 bp |
| MMP9 anti-sense | CAGGTGACGGGCTGCTT |  |
| Bmp2 sense | ACGTCCTCAGCGAATTTGAG | 239 bp |
| Bmp2 anti-sense | TTTCCCACTCATCTCTGGAAGT |  |
| β-actin sense | CCTGAGGCTCTTTTCCAGCC | 120 bp |
| β-actin anti-sense | TAGAGGTCTTTACGGATGTCAACGT |  |





**Figure S1.** Immunofluoresce of BMP2 (marker of decidulization) protein with decidual cells isolated from decidual tissue.

© 2015 by the authors; licensee MDPI, Basel, Switzerland. This article is an open access article distributed under the terms and conditions of the Creative Commons Attribution license (http://creativecommons.org/licenses/by/4.0/).
